# Advances and current concepts on Eph receptors and ephrins in upper digestive tract cancers

**DOI:** 10.3389/fonc.2024.1520306

**Published:** 2025-01-07

**Authors:** Luccas Lavareze, Talita de Carvalho Kimura, João Figueira Scarini, Reydson Alcides de Lima-Souza, Moisés Willian Aparecido Gonçalves, Raisa Sales de Sá, Iara Gonçalves Aquino, Patricia Maria Fernandes, Fernanda Cristina Poscai Ribeiro, Albina Altemani, Fernanda Viviane Mariano, Gary Chris Fillmore, Erika Said Abu Egal

**Affiliations:** ^1^ Department of Pathology, Faculty of Medical Sciences, University of Campinas (UNICAMP), Campinas, São Paulo, Brazil; ^2^ Department of Oral Diagnosis, Piracicaba School of Dentistry, University of Campinas (FOP/UNICAMP), Piracicaba, São Paulo, Brazil; ^3^ Department of Internal Medicine, University of Western São Paulo (UNOESTE), Guarujá, São Paulo, Brazil; ^4^ Biorepository and Molecular Pathology, Huntsman Cancer Institute, University of Utah (UU), Salt Lake City, UT, United States

**Keywords:** Eph, gastric cancer, target therapy, upper gastrointestinal, ephrin

## Abstract

Erythropoietin-producing hepatocellular (Eph) receptors comprise the largest group of surface receptors and are responsible for cellular signals. Eph/ephrin signaling has been identified to play a role in key cancer development and progression processes, especially in the upper gastrointestinal tract. The Eph/ephrin system has been described as a tumor suppressor in duodenal cancer, while in esophageal, gastric, hepatic, and pancreatic cancer, the system has been related to tumor progression. For their significant role in developing a wide range of malignancies, Eph receptors and their ligands have proven to be an important target for new anticancer therapies. In this review, we present an overview of the literature and highlight evidence supporting the role of the Eph/ephrin system in upper digestive tract cancers. In addition, we discuss molecular findings that represent promising therapeutic targets for these cancers.

## Graphical Abstract

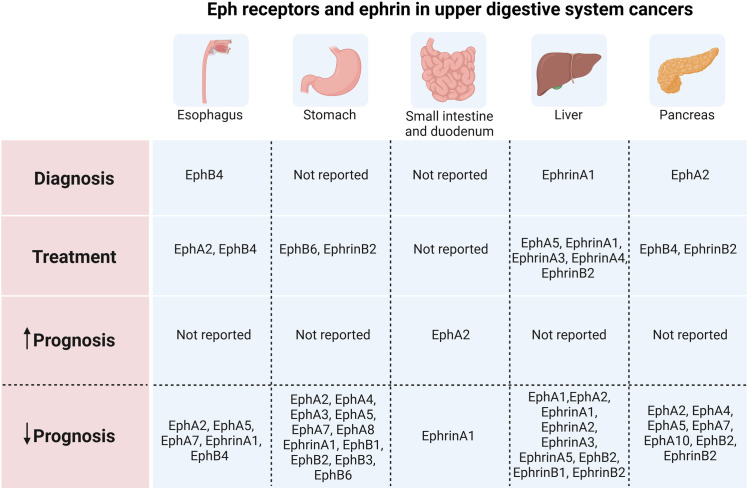

## 1 Introduction

Upper digestive cancer (UDC) is a class of cancers affecting the esophagus, stomach, and small intestine. As the global cancer burden continues to rise, gastrointestinal (GI) tract tumors, including those originating in the stomach, colorectum, and liver, rank among the five most prevalent cancers worldwide in men whereas stomach and liver tumors are the leading causes of cancer-related deaths (1). In women, colorectal cancer is the second most frequently diagnosed cancer, followed by stomach cancer (1).

The Eph (erythropoietin-producing hepatocellular carcinoma) receptor family, the largest subgroup of tyrosine protein kinase receptors, plays a crucial role in normal physiological function as well as disease. This is achieved through their interaction with ephrin, which generates bidirectional signals at sites of cell-cell contact (2). For its significant role in developing a wide range of malignancies, Eph receptors and their ligands proved to be an important target for new anticancer therapies. Over the years, several drugs have been developed that target Eph/ephrin signaling, including dasatinib, sitravinib, JI-101, XL647, and ifabotuzumab (3). Except for Ifabotuzumab, these drugs demonstrate a multikinase effect and were not developed primarily as an anti-Eph drug, making the results of the studies conflicting regarding the direct effect on the molecule. So far, there are no clinical trials specifically targeting Eph receptors in UDC, but there are promising results in lung cancer and leukemia (4, 5). With the increased knowledge about the Eph/ephrin system and its potential role in tumorigenesis, Eph receptors and their ligands may be more common in the current therapies for cancer treatment. Given the conflicting data regarding the roles of Eph receptors in UDC, here we aim to underscore evidence that highlights the complex and often ambiguous functions of the Eph/ephrin system in these tumors.

## 2 Overview

### 2.1 The role of EPH receptors and ephrin in cancer

Among the 20 receptor tyrosine kinase (RTK) subfamilies, erythropoietin-producing hepatocellular (Eph) receptors comprise the largest group of surface receptors (3, 6). They are two subfamilies of Eph receptors, the EphA subclass, consisting of nine members (EphA1-A8 and EphA10), and the EphB subclass, composed of five members (EphB1-B4 and EphB6) (3, 7). The classification of each Eph receptor depends on the types of ligands (ephrins) that they bind. The ligands of the Eph receptors are divided into two subclasses: Ephrin A ligands, which are attached to the cell surface by a glycosylphosphatidylinositol anchor, and Ephrin B ligands, which contain a transmembrane domain and a short cytoplasmic region (3, 7). A-type receptors typically bind to most or all A-type ligands, and B-type receptors bind to most or all B-type ligands (8). The exception includes EphA4, which can bind both A-type and most B-type ligands, and ephrin A5, which can bind to EphB2 in addition to A-type receptors, but not to other EphBs (8, 9).

**Figure 1:**
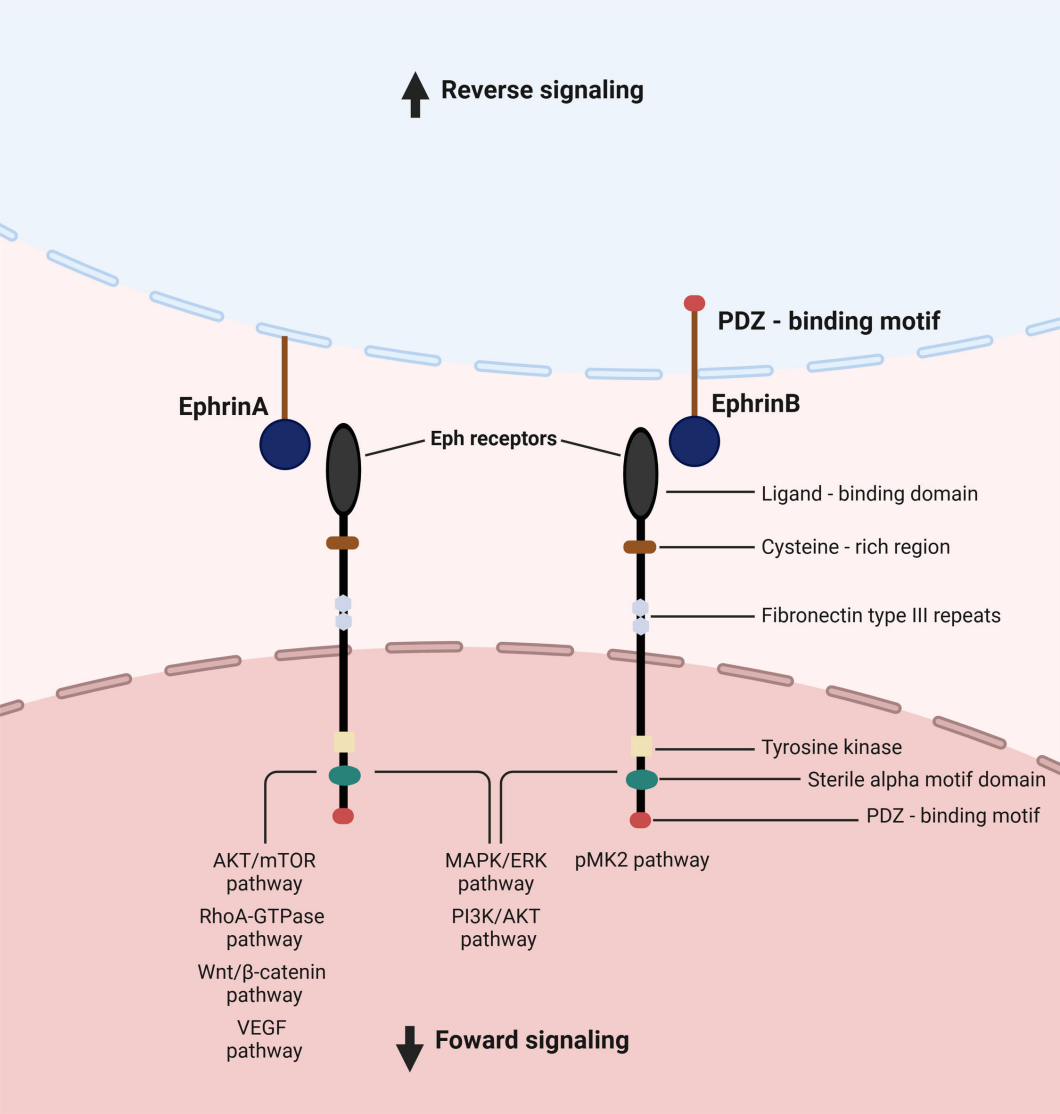
Eph signaling, and pathways involved in upper digestive tract cancers. The interaction between Eph receptors and ephrin ligands constitutes a crucial cellular communication system characterized by bidirectional signaling, encompassing both forward and reverse pathways. Ephrin ligands possess the ability to function as receptors, while Eph receptors can also serve as ligands. “Forward signaling” primarily operates via phosphoserine-dependent pathways. Within this forward signaling mechanism, various molecular cascades are triggered to propagate the signal intracellularly. In “reverse signaling,” signal transduction occurs within the cell expressing ephrins.

The interaction between the Eph receptors and ephrin ligands forms an important cell communication system with bidirectional signaling, reverse and forward. An ephrin ligand can function as a receptor, and an Eph receptor can also act as a ligand (9). The so-called ‘forward signaling’ mostly occurs through phosphoserine-dependent pathways (6). In this forward signaling, a variety of molecular cascades are activated to further convey the message into the cytoplasm, such as Janus kinase (JAK)-signal transducer and activator of transcription (STAT), GTPases of the Rho and Ras family, focal adhesion kinase (FAK), as well as the phosphoinositide 3-kinase (PI3K) (8). In reverse signaling, the signal transduction occurs in the ephrin-expressing cell. For example, the activation of signaling effectors and the initiation of a signal transduction cascade are caused by the phosphorylation of tyrosine residues in the cytoplasmic tail of B-ephrins (9) (**Figure 1**).

The interactions of the Eph/ephrin system promote extensive roles in normal physiology and disease pathogenesis. For instance, the interaction of Eph with the Src family kinases results in the regulation of synapse formation, and Rho GTPases lead to junction stabilization. Additionally, the interaction with ERK/MAPK leads to cell proliferation and the interaction with FAK and JAK/STAT results in modulation of cell adhesion (8). During embryogenic development, where their expressions are more prominent, they can regulate cell movement and adhesion during gastrulation, somatogenesis, tissue, and organ boundary formation, axon guidance, vascular development, and other developmental processes (8, 10).

Eph receptors and ephrins are less active in adulthood, but they still perform crucial roles, such as stem cell function as well as in tumorigenisis (11). The significance of Eph/ephrins signaling in carcinogenesis and the potential for metastasis in relation to the growth and survival of tumors is one of the main effects of these molecules (9). The expression of Eph receptors and their ligands is frequently conflicting, and the underlying molecular pathways are complex in diverse malignancies. Interestingly, Eph receptors can either stimulate or restrain tumor growth in malignant tumors (3). Numerous studies have demonstrated the role of the Eph/ephrin system in a variety of cancers, including carcinoma from the gastrointestinal tract (12–14), colon (15, 16), pancreas (17), esophagus (18, 19), liver (20), prostate (21, 22), lung (23, 24), thyroid (25), breast (26, 27), ovaries (28), and others.

Eph/ephrin signaling has been identified to play a role in key processes that are known to be significant in tumorigenesis and metastasis. For instance, many of VEGF’s angiogenic functions have been found to be inhibited by EphA2 (29). Blocking the EphA receptor may inhibit VEGF-dependent endothelial cell migration, growth, survival, and angiogenesis (9). In addition, the stimulation of EphA2 reduces FAK phosphorylation, resulting in the inhibition of integrin-mediated cell adhesion (30). Integrins have a direct impact on cell motility and invasion because of their critical function in mediating cell anchoring, adhesion, and fibronectin deposition (9). As a result, they aid in the growth of malignancies and metastasis, contributing to carcinogenesis (9).

## 3 Eph receptors and ephrin in upper digestive system cancers

### 3.1 Esophageal tumors

Esophageal cancer is the seventh most common malignant tumor contributing to half a million deaths in 2020 worldwide (1). Esophageal squamous cell carcinoma (ESCC) is the predominant histologic subtype, representing more than 90% of cases in developing countries. ESCC is followed by esophageal adenocarcinoma (EAC) which is even more prevalent in developed nations, reaching two-thirds of cases (1, 31, 32). While tobacco use and alcohol consumption are the major risk factor for SCC, EAC is related to gastroesophageal reflux disease and obesity settings (31). Multimodality treatment, involving surgery, chemo, and radiotherapy is the major therapeutic approach and, despite these efforts, 5-year overall survival remains around 20% (1).

Several Eph receptors and ephrin ligands have been identified in tumor samples of esophageal cancers from patients or cell lines and their levels of expression have been correlated with the progression of cancer (**Table 1**). However, the mechanisms of direct regulation of Eph remain unknown and further studies are needed to verify the role of these receptors in the progression of esophageal cancer, especially in EAC, where studies are still scarce.

**Table 1:** Eph expression and role in esophageal cancer .

Author	Year	Eph receptors/ephrin ligands	Study Method	Esophageal histologic subtype	Main findings
Zhang et al. (33)	2020	EphA5	IHC; *in vitro* assay	ESCC	EphA5 knockdown trigged EMT by activating Wnt/β-catenin signaling in ESCC.
Chen et al. (34)	2018	EphA3	*In vitro* assay	ESCC	Expression levels of EphA3 were decreased in ESCC. Overexpression in ESCC cells led to EMT and inhibited cell migration and invasion.
Bai et al. (19)	2015	EphA7	IHC	ESCC	EphA7 expression is involved in the differentiation and lymph node metastases of ESCC.
Syed et al. (35)	2015	EphA2	*In vitro* assay	ESCC	Knockdown of EphA2 in ESCC cell line TE8 resulted in a significant decrease in cell proliferation and invasion.
Miyazaki et al. (36)	2005	EphA2	IHC	ESCC	Overexpression of EphA2 was correlated with disease advanced stage.
Xu et al. (37)	2005	EphA2/EphrinA-1	IHC; *In vitro* assay	ESCC	Both EphA2 and ephirinA1 were related to poor overall survival. EphA2 was considered an independent prognostic marker.
Miyazaki et al. (18)	2003	EphA2	IHC	ESCC	EphA2 overexpression was correlated with poor tumor differentiation and regional node metastasis. Additionally, EphA2 positive patients presented lower survival rates.
Tachibana et al. (38)	2007	EFNB2 ligand and EphB4	IHC; *in vitro* assay	ESCC	The results revealed positive correlations between EFNB2 expression and the number of lymph node metastases and stage of the disease.
Hasina et al. (39)	2013	EphB4	IHC; *In vitro* assay; *In vivo* models	ESCC/EAC	EphB4 expression was higher in ESCC and AEC in adjacent normal tissue. EphB4 contributes to tumor biology, being involved with increased proliferation, motility, and migration of cancer cells.

EAC, esophageal adenocarcinoma; EMT, epithelial-mesenchymal transition; ESCC, esophageal squamous cell carcinoma; IHC, Immunohistochemistry.

#### 3.1.1 Ephrin-A and EphA

The first association between Eph receptors and esophageal cancer was reported in 2003 by Miyazaki et al. The authors found through an IHC study that the overexpression of the EphA2 protein was correlated with poor tumor differentiation and regional lymph node metastasis. The authors also found that EphA2 was positively expressed in fifty percent of the studied population, presenting survival rates significantly lower than in EphA2-negative patients. Complementing these findings, they identified by western blotting that Eph2 was more expressed in ESCC cell lines than in untransformed cell lines, suggesting that the level of the EphA2 protein reflects the tumor’s malignancy (18).

Corroborating with the above-cited results, a further investigation performed by the same authors found that the overexpression of EphA2 was correlated with the advanced stage of the disease. Such results suggested that EphA2 expression levels could be assessed before the treatment approach (36).

The ephrinA1 ligand and EphA2 are co-expressed in various types of human malignant tumor cells such as in breast cancer and Kaposi’s sarcoma, influencing tumor neovascularization (40). Analyzes of EphA2 and ephrinA1 protein and mRNA expressions in ESCC samples revealed that both markers are co-located in the same tumor areas and vascular endothelial cells. Thus, tumors with positive immunostaining had higher mRNA levels than those with negative staining. Noteworthy, no difference was observed regarding the intensity of staining in the positive cases, indicating that the expression of mRNA did not fully correspond to its protein expression in ESCC (37). In addition, high levels of EphA2 and ephrinA1 expression were significantly associated with lower overall survival, making EphA2 a strong independent predictor that could be used as a prognostic marker for ESCC (37).

To better understand the above-cited findings, a study of the phosphoproteomic profile in ESCC cell lines was performed to identify the tyrosine kinase signaling pathways activated in this cancer. A total of 278 unique phosphopeptides were identified and *EPHA2* was hyperphosphorylated in all studied ESCC cell lines. Next, *EPHA2* siRNA-mediated knockdown was performed showing high hyperphosphorylation, resulting in a significant decrease in cell proliferation and invasion (35). Together, this evidence strongly suggests that *EPHA2* acts as an oncogene in the ESCC, becoming a promising therapeutic target, which justifies in-depth mechanistic studies in additional *in vitro*, transgenic, and PDX mouse models.

However, researchers have suggested that other members of the EphA family may have a tumor suppressor role in the ESCC. A previous IHC study on EphA7 protein expression in 352 cases of ESCC showed that the low expression of EphA7 was significantly associated with lymph node metastasis, low degree of tumor differentiation, and pTNM staging. In addition, the low expression of EphA7 was correlated with a lower survival rate, although it was not considered an independent prognostic factor (19). In this sense, previous studies have shown in colorectal cancer (41), gastric carcinoma (42), and prostate cancer (43) that downregulation of EphA7 may also play an important role in carcinogenesis and differentiation. Nevertheless, EphA7-specific signal transduction pathways mediating carcinogenesis in ESCC have not yet been elucidated.

Studies on EphA3 also suggested that this protein may act as a tumor suppressor in ESCC. Chen et al. (2018) detected lower levels of *EPHA3* mRNA expression in ESCC tissues compared to the normal counterparts, while in cell lines EphA3 expression was minimal or undetectable. These findings were in agreement with previous studies, which revealed that the *EPHA3* gene is deleted in the ESCC (44, 45). Also, the knockdown of EphA3 induced EMT and promoted cell migration and invasion via the RhoA-GTPase signaling pathway (**Figure 1**). Interestingly, the authors concluded that EphA3 expression was significantly suppressed in the ESCC due to promoter methylation. Additionally, EphA3 functions were dependent on kinase activity and tyrosine phosphorylation status (34). Recently, Zhang et al. (2020) demonstrated that EphA5 acts as an EMT suppressor by activating the Wnt/β-catenin pathway and therefore plays an essential role in ESCC migration and invasion. The authors detected high expression of EphA5 in ESCC tissues and low expression in normal esophageal epithelial tissues. Further analyses showed that high EphA5 expression was correlated with the presence of regional metastasis and advanced-stage disease, while low expression of this receptor was associated with neural invasion (33). In this study, the functions of EphA5 and its molecular mechanisms were also investigated in ESCC cell lines. The knockdown of EphA5 increased malignant characteristics of cells *in vitro*, such as the capacity for cell proliferation, migration, and invasion, as well as induced EMT. It is worth mentioning that the high expression of EphA5 correlated with metastases in lymph nodes in patients with ESCC although this seems to be inconsistent with the results *in vitro*. Therefore, it is necessary to investigate the effect of EphA5 on other signaling pathways that contribute to regional lymph node metastasis (33).

In summary, while EphA2 promotes cancer progression in ESCC, EphA7, EphA3, and EphA5 act as tumor suppressors, suggesting that members of the Eph family may have opposing effects depending on the tumor context.

#### 3.1.2 Ephrin-B and EphB

Bioinformatic analyzes using the Oncomine platform revealed a dramatic increase in EphB1, EphB2, and EphB4 in esophageal cancer, suggesting these receptors as potential biomarkers (46). Among the four members of the Eph family B receptors, only one subtype has been identified in ESCC to date. EphB4 has been reported to have tumor-suppressive properties in breast cancer (47). However, in esophageal cancer, its overexpression is related to tumor progression. The first study evaluating gene and protein expression of the *EFNB2* ligand and its EphB4 receptor in patients with ESCC showed correlations between high EFNB2 expression, advanced stage of the disease, and a worse prognosis (38). Another study by Hasina et al. (2013) showed that EphB4 expression was consistently higher in squamous cancer and adenocarcinoma than in adjacent normal tissue. Additional results revealed that EphB4 contributes to increased proliferation, motility, and migration of cancer cells. Moreover, in a tumor cell xenograft model, a significant reduction in tumor volume was observed after treatment with an EphB4 inhibitor, reinforcing its effectiveness as a new biomarker and molecular target for esophageal cancer (39).

### 3.2 Gastric tumors

Gastric cancer (GC) is the sixth most common cancer worldwide and the type of cancer fourth most related to death (48). Despite the decline in cancer-related mortality in recent years, the GC prognosis remains uncertain (49). Most GC are adenocarcinomas that arise from glands in the superficial layer of the stomach (50) and may be related to poor diet, smoking, alcoholism, and *Helicobacter pylori* infection (50, 51). Besides the known risk factors, GC may be related to several local and genetic factors that act collectively during carcinogenesis (52). Surgery remains the only treatment with curative purpose while QT and RT are reserved for advanced tumors (53). The role of Eph-Ephrin in GC progression has been questioned for over twenty years (54), being responsible for a massive literature development in this field.

#### 3.2.1 Ephrin-A and EphA

**Figure 2:**
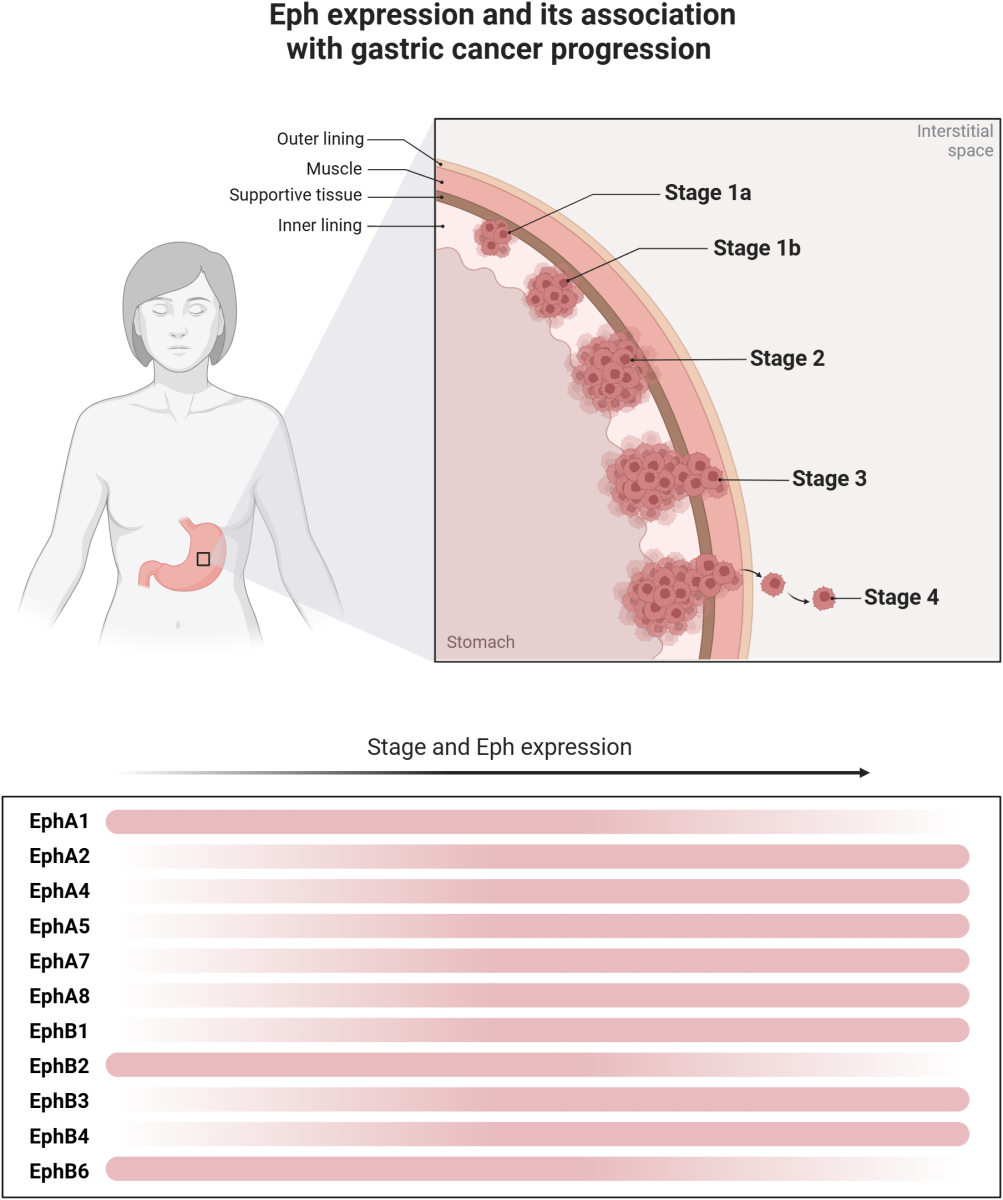
Eph expression and its association with gastric cancer progression. Different levels of Eph expression are observed throughout the progression of gastric cancer. The figure illustrates the dynamics of the expression of these molecules along tumor progression, revealing a dynamic change in the expression gradient, represented by the transition of the red bars. In the early stages of tumor development and differentiation, greater expression of EphA1, EphB2 and EphB6 is observed. However, in more undifferentiated tumors and with the progression of the disease stage, there is greater expression of EphA2, EphA4, EphA5, EphA7, EphA8, EphB1, EphB3 and EphB4, which then contribute to poor disease behavior.

EphA1 and ephrinA1 are up or downregulated in several malignant tumors, such as advanced skin cancer and colorectal cancer (52). In GC, the *EFNA1* and ephrinA1 receptor polymorphisms were related to susceptibility to tumor development (55). Also, ephrinA1 was related to worse clinical staging, lymph node metastasis, and poor prognosis (56–59)(**Figure 2**). Furthermore, the knockout EphA1 from GC cell lines reduced invasiveness *in vivo* showing a potential role in tumor progression (58). Nevertheless, EphA1 was detected more frequently in well-differentiated tumors indicating its role in the differentiation of GC (60). Zhuo et al. (2019) described a lncRNA (*GMAN*) overexpressed in GC that binds competitively with *EFNA1*. The author concluded that targeting both genes may be an important therapeutic option for GC treatment (58).

EphA2 is seen to also play a significant role in the GC progression (56, 61) (**Figure 2**). GC with EphA2 overexpression may show a greater possibility of invisible metastasis (62), distant metastasis (63), tumor invasion, worse clinical staging (51, 56, 60, 64, 65), recurrence (62), tumor differentiation (60), and poor prognosis (64). Also, the expression of EphA2 was associated with proliferation, colony formation, migration, and cell motility *in vitro* (61, 63, 65, 66). The EphA2 mechanism of carcinogenesis is still unknown, however, the EphA2 expression seems to be a driver of EMT via Wnt/β-catenin pathway (**Figure 1**) (63, 67). A study conducted by Huang et al. (2017) showed the inhibition of the EphA2 via Wnt/β-catenin by activation of miR-302b presented an important antitumor activity *in vitro* and in a mouse xenograft model (male BALB/c nude mice), followed by decreasing the cell growth, migration, and metastasis (66). EphA2 also seems to present a role in drug resistance. *In vitro* studies showed that *EPHA2* overexpression was related to increased tumor cell resistance to 5-FU and afatinib, a multi-kinase inhibitor (61, 68). EphA2 seems to induce phosphorylation of yes-associated protein (YAP), a transcriptional coactivator that promotes cell proliferation and tissue homeostasis, which may be the mechanism related to tumor drug resistance (61). Interestingly, the bacteria *H. Pylori* seems to target EphA2 (51).

EphA3 expression is seen to be related to tumor progression by angiogenic activity in GC (69, 70). EphA3 was associated with vascular endothelial growth factor (VEGF) expression and MVD quantification in those tumors (70). Studies show that EphA3 depletion inhibited cell growth and angiogenesis of GC cells *in vitro* and *in vivo* (69, 70). Also, EphA3 overexpression was associated with histological differentiation, tumor depth, lymph node metastasis, distant metastasis, and poor prognosis (12) (**Figure 2**). Similar tumor behavior and clinical course were found with the overexpression of EphA4 (71), EphA5 (72), and *EPHA8* (73). EphA7 seems to play a carcinogenic role in younger patients and was related to advanced tumors (42) (**Figure 2**).

In summary, most Eph receptors (EphA1, EphA2, EphA3, EphA4, EphA5, and EphA7) function as promoters of GC progression, influencing metastasis, tumor invasion, angiogenesis, and drug resistance, while EphA1 has a more complex role depending on tumor differentiation.

#### 3.2.2 Ephrin-B and EphB

In gastric tissue, ephrinB1 is highly expressed in the superficial gastric regions, more specifically in the pit cells of the body and the proliferating cells of the isthmus (74). The expression of EphB1, EphB2, and EphB3 is higher in deeper regions of the gastric units (74). Besides the EphB in normal gastric mucosa, the combined expression of EphB2, B3, and B4 with ephrinB1 was associated with gastric dysplasia (75), showing that EphB-ephrin-B dysfunctions may contribute to tumor development and progression (74) (**Figure 2**).

EphB1 is a member of the receptor tyrosine kinase that plays important role in angiogenesis (76). EphB1 also appears to exhibit a tumor suppressor role in GC. Studies indicate that low expression of EphB1 is associated with disease stage, invasion, and metastasis (77) (**Figure 2**). Tanaka et al. (2010) used amino acids derived from EphB1 composition to treat scirrhous GC cells. The combination inhibited RhoA activation and extracellular metalloproteinase-8 (MMP-8) secretion. Application of the ephrinB1 peptide to mouse peritoneum suppressed carcinomatous peritonitis, showing the potential for its use as therapy (78). On the other hand, blocking phosphorylation of ephrinB1 decreased cancer cell dissemination and local invasion in a mouse xenograft model (BALB/c nude mice), indicating a tumor-promoting activity (79). Also, *EPHB1* overexpression was more frequently detected in poorly differentiated adenocarcinoma (14).

EphB2 is one of the most studied Ephs in malignant tumors (80). In GC, EphB2 overexpression has been reported in tumor (14, 54, 81) and serum samples (13). The first relationship of EphB2 in GC was reported in 1994, attributing its overexpression to chromosomal locus 1p36, generally considered the tumor suppressor locus of colon cancer (54). Several studies showed that EphB2 may present the same role in GC carcinogenesis (82) (**Figure 2**). *EPHB2* downregulation was related to nodal metastasis, advanced disease stage, low histological differentiation, and poor survival (14, 83). Furthermore, it was noted that tumor grade increased as EphB2 levels decreased (83). On the other hand, EphB2 activation was related to high levels of migration and invasion *in vitro* (81) along with tumor size, metastasis, clinical staging (13), and poor survival (13, 84). Nevertheless, further studies showed an association between EphB2 and better survival (85). Overall, EphB2 seems to act at some point in GC carcinogenesis and may be explored as a potential therapeutic target (84, 85).

Similarly, EphB3 appears to have tumor suppressor activity. Depletion of *EPHB3* promoted cell growth and invasion *in vitro* while EphB3 downregulation is associated with increased tumor size, invasion, clinical staging, lymphatic metastasis, and poor prognosis (**Figure 2**) (86). On other hand, *EPHB3* upregulation seems to be related to resistance to tyrosine kinase inhibitors such as AZD4547 (87).

EphB6 is poorly described in the GC literature and may be a potential metastasis inhibitor (88). The EphB6 expression was detected in four GC cell lines, its overexpression was associated with tumor differentiation while its underexpression was associated with lymph node metastasis and tumor stage (**Figure 2**) (88).

To summarize, EphB receptors play tumor suppressor and promoter roles. EphB1 and EphB3 act as tumor suppressors, and their low expression is associated with metastasis and a poor prognosis. EphB2 has a complex role, in which its overexpression is associated with tumor progression, and downregulation correlates with worse outcomes. EphB6 can inhibit metastasis, and its downregulation is associated with advanced disease. Thus, EphB receptors can suppress or promote tumor progression, depending on their expression in the GC.

### 3.3 Small intestine

The small bowel includes the duodenum, jejunum, and ileum (89). Small bowel tumors are rarer than other cancers of the gastrointestinal tract (90). This may explain the difference in research aimed at the Eph/Ephrin interaction in these tumors when compared, for example, with colon carcinomas (91).

The epithelium of the small intestine presents an accelerated cellular turnover rate, associated with significant structural and functional differentiation (92). This aspect may explain the expression heterogeneity of these tyrosine kinases in the small intestine and the role of Eph/Ephrin signaling in the regulation of the cellular positioning of the pyloric and duodenal epithelium. Ephrins are preferentially expressed in the superficial part (such as cells lining the ducts of the Brunner glands, as well as those covering villi and the upper portion of lieberkühn crypts) while EphB receptors are most expressed in the deepest part of epithelial tissue (such as in the segment of the Brunner glands and the lower portion of the crypts) (93).

Islam et al., 2010 showed that EphA4, EphA8, EphB4, and ephrinB2 were more expressed during the fetal period, and may be able to regulate the migration of intestinal epithelial cells during morphogenesis (92). This pattern of expression seems to remain in the stages after development, except for EphA8, whose expression is not seen in normal adult intestinal tissue (92, 94). Co-expression of EphB2 and ephrinB1/B2 in the normal bowel mucosa is mainly related to cellular homeostasis (95). Recently, Zhu et al. (2021) reported that the maintenance of this homeostasis may be through *Notch* signaling (96) or via β-catenin/TCF dependent, contributing to the ordering of cellular populations through Eph/Ephrin signaling (97). Data in the literature show that EphB receptors may be important regulators of migration and proliferation also of intestinal stem cells (ISCs) (98–100) and this may provide important insights into therapy for small intestinal tumors.

A robust study evaluated the expression of EphB2 protein in a cohort of 138 different types of cancer. In the small intestine, EphB2 protein expression was found in 70% of small bowel adenocarcinomas (82). Bogan et al. (2009) showed that the gene and protein expression of EphA2 was significantly regulated in small intestine tumors when compared with control tissues. Genetic knockout of the *EPHA2* gene in rats has been translated into reducing the number and size of tumors in both the small and large intestines (101). Regarding the expression pattern of ephrins, the increased expression of ephrinA1 accelerated the process of malignant transformation of small and large intestinal adenomas (102).

In summary, Eph/Ephrin signaling in small bowel tumors exhibits both tumor-promoting and tumor-suppressing roles. EphB receptors, particularly EphB2, are crucial for maintaining cellular homeostasis and regulating stem cell function, while EphA2 and ephrinA1 contribute to tumor progression and malignant transformation. These findings highlight the complex and context-dependent roles of Eph/Ephrin signaling in developing small bowel tumors.

### 3.4 GI glandular organs

#### 3.4.1 Liver tumors

Approximately 80% of primary liver malignancies constitute hepatocellular carcinomas (HCCs). About 906,000 cases of HCCs were diagnosed worldwide in 2020 (103, 104). HCC predominantly impacts the male population and tends to manifest within individuals aged 60 to 70 years (105). Additionally, this tumor is the third main contributor to global cancer-related fatalities, exhibiting a 5-year survival rate of approximately 18% (106). Notably, persistent liver disease arising from chronic infections with hepatitis B virus (HBV) or hepatitis C virus (HCV), excessive alcohol consumption, and the presence of non-alcoholic fatty liver disease (NAFLD) or non-alcoholic steatohepatitis (NASH) are the primary precursors to HCC (105, 107).

The second most common type of liver cancer is cholangiocarcinoma (CCA), which predominantly affects the bile ducts, originating from cholangiocytes (108). In recent decades, CCA has shown an increase from 0.1 to 0.6 cases per 100,000 people (105). As treatment, for early-stage CCA, surgical resection associated with chemotherapy is the first option. However, many patients are diagnosed at late stages and the prognosis becomes worse (109). The Eph/ephrin signaling network has emerged as a viable candidate for therapeutic intervention in liver cancer. Modulating this intricate system holds substantial promise as a strategy to tackle liver cancers, encompassing both HCC and CCA (107).

##### 3.4.1.1 Ephrin-A and EphA

The role of EphA1 in angiogenesis and HCC progression has been investigated. It was shown that suppression of EphA1, in an *in vitro* model, led to a reduction in proliferation, motility, and invasive capacity of HCC cells (110). A correlation was also seen between hypoxic conditions and elevated ephrinA1 expression in HCC cell lines (PLC/PRF/5, HuH7, HepG2 and Hep3B cells) (111). Alpha-fetoprotein (AFP) plays the role of a wide-ranging biomarker in the detection and monitoring of HCC. Elevated serum AFP levels signal a consistent association with unfavorable prognosis for HCC, giving it significant relevance. In this sense, ephrinA1 expression was elevated in HCC samples and strongly correlated with AFP expression, suggesting a contribution to the malignant characteristics of AFP-producing HCC. It also promotes tumor cell growth, angiogenesis, invasion, and metastasis (112). Therefore, it is suggested that molecules of the ephrinA1 system may play a crucial role in the pathogenesis and progression of AFP-associated HCC and may serve as biomarkers of the disease (112).

Studies in animal models have demonstrated that EphA2 is critically important for tumor growth in HCC. Furthermore, suppression of tumor initiation against EphA2 using CRISPR-Cas9 mediated increased overall mouse survival (113, 114). Regarding CCA, EphA2 is seen to play an important role in its pathogenesis, since EphA2 is overexpressed in response to growth factors, leading to activation of the mammalian target of rapamycin complex 1 (mTORC1) and extracellular signal-regulated kinase (ERK) pathways (**Figure 1**). This overexpression facilitates tumor growth by activation of Akt (T308)/mTORC1 (115). EphrinA2 was shown to be up-regulated in HCC cell lines and clinical tissue samples in tumors invading the portal veins. Overexpression of ephrinA2 in HCC cells were found to increase tumorigenicity *in vivo*, while its suppression had the opposite effect (116).

When investigating the levels of eNOS and phosphorylated eNOS (P-eNOS), together with the regulators VEGFR3, VEGFC, EphA3 and ephrin1, in a hamster model of induced CCA and human CCA, it was seen that in the latter intense immunohistochemical staining of all examined proteins was associated with metastasis. Therefore, up-regulation of eNOS, P-eNOS and their regulators is involved in CCA development by potentiating angiogenesis and metastasis (117). It has been seen that *EFNA3* signaling induced by miR-210 may be a potential target of cisplatin in the treatment of HCC (118). When studying the role of ephrinA3 in hypoxia, it was seen that there is an up-regulation of ephrinA3 by hypoxia in a HIF-1α dependent manner in HCC tumors, which was associated with worse overall survival (119).

When investigating the role of ephrinA4 in HCC it was found that this ligand was highly expressed in HCC cell lines and its inhibition significantly reduced cell proliferation, migration, and invasion in Huh7 cells. In addition, another study conducted both *in vitro* and in a mouse xenograft model suggested that ephrinA4 may serve as a potential therapeutic target for this tumor (120).

Studies using human umbilical cord-derived mesenchymal stem cells suggested that EphA5 can be investigated as a biological therapy for HCC (121). The co-activation of ALK, FGR2 and EphA5 function as central kinases in HCC cells and this co-activation is important for cell growth. In this sense, the presence of this triple-positive state can be used as a potential therapeutic target for treatment purposes (122). When exploring the regulation of *EFNA5* expression in HCC using miR-96 and miR-182, a reciprocal correlation between the expression levels of these miRNAs and the levels of ephrinA5 was observed and associated with increased proliferation and migration of HCC cells (123).

In summary, suppression of EphA1 in models of HCC resulted in reduced proliferation, motility, and invasion of tumor cells. EphA2 and ephrinA2 play essential roles in HCC growth and invasion, with potential as therapeutic targets. The inhibition of ephrinA3 and ephrinA4 has shown promise in reducing the proliferation, migration, and invasion of HCC cells, suggesting a therapeutic potential. In addition, EphA5 and its coactivation with ALK and FGFR2 also represent potential targets for biological therapy and treatment of this type of tumor.

##### 3.4.1.2 Ephrin-B and EphB

*EFNB1* was highly expressed in HCC tissues compared to normal samples, and high expression of this gene was associated with tumor progression and vascular invasion, which led to a poor prognosis in HCC patients (124). Also, Sawai et al. demonstrated that overexpression of ephrinB1 is involved in HCC progression by promoting neovascularization *in vivo* (125).

To understand the mechanisms related to tumor and endothelial cells, crosstalk between HepG2 cells (human hepatocellular carcinoma, HCC) and human endothelial progenitor cells (EPCs) in co-culture was investigated. The results showed that both Delta-like 4 (DLL4), a protein related to angiogenesis, and ephrinB2 appear to play essential roles in various stages of this activity. The high expression of ephrinB2 and DLL4 associated with the activated phenotype of EPCs suggests their involvement in the development of HCC. In summary, these findings highlight ephrinB2 and DLL4 as promising targets for new clinical strategies for the treatment of this tumor (126).

An *in vivo* and *in vitro* study evaluating the effects of HMQ-T-B10 (B10) on HCC. In this study, it was found that ephrinB2 is overexpressed in liver cancer and can serve as a promoter of apoptosis. B10 has an inhibitory effect on HCC cells, by targeting the ephrinB2 signaling pathway and inducing apoptosis. Thus, B10 represents a promising anticancer agent for new clinical trials for the treatment of liver cancer (127).

Regarding the role of these ligands in CCA, Khansaard et al. studied their importance and association with metastasis. Upon evaluation of immunohistochemical staining in 50 cases of CCA, high expression of EphB2, EphB4, ephrinB1 and ephrinB2 was seen. Regarding metastasis, high expression of EphB2 and co-expression of EphB2/ephrinB1 and EphB2/ephrinB2 were seen to be associated with metastasis. In summary, the increased expression of EphB2 receptors along with their corresponding ligands (ephrinB1 and ephrinB2) is associated with CCA metastasis. Potential therapeutic strategies for this tumor may involve targeting EphB2 expression and inhibiting its downstream signaling proteins (128).

To summarize, high expression of EphrinB1 and EphB2 play significant roles in the progression of HCC and CCA. High expression of ephrinB1 in HCC is associated with tumor progression, vascular invasion, and poor prognosis. EphrinB2 is also overexpressed in HCC, promoting angiogenesis and apoptosis inhibition, making it a potential target for anticancer therapies. In CCA, EphB2 and its ligands (ephrinB1 and ephrinB2) are linked to metastasis, suggesting that targeting EphB2 and its signaling pathway could be a promising strategy for treating CCA.

#### 3.4.2 Pancreatic tumors

Pancreatic cancer (PC) is the 12th most common malignant tumor worldwide (129, 130). PC has a poor prognosis where 94% of patients do not achieve a 5-year survival and 74% succumb within a year of diagnosis, consolidating its status as one of the most lethal cancers (130). PC are classified as either exocrine or neuroendocrine tumors, depending on their cellular origin. Among these, pancreatic ductal adenocarcinoma (PDAC) and pancreatic neuroendocrine tumors (PanNETs) are the major types, with PDAC accounting for approximately 90% of cases (131, 132).

The EPH/ephrin system has been linked to several processes that pertain to the embryological integration of the pancreatic parenchyma and the arrangement of the islets of Langerhans (133). These islets are a significant endocrine component responsible for orchestrating the regulation of insulin secretion (134). The EPH/ephrin system’s Class B molecules play a pivotal role in orchestrating pancreatic morphogenesis, mainly pancreatic epithelial alignment, branching of structures and lumen formation, by the interaction between EphB2 and EphB3.

##### 3.4.2.1 Ephrin-A and EphA

Regarding the role of Eph/Ephrin in PDAC, EphA2 and EphA4 are the most important targets in the field of translational research (135). EphA2 has attracted the attention of the research community due to its involvement in tumor capillary formation (40). Van den Broecket et al. presented compelling evidence showcasing the marked overexpression of EphA2 within the context of PDAC, a phenomenon that correlates with adverse clinical outcomes (136). Furthermore, EphA2 has also emerged with clinical significance as a biomarker, with results showing an impressive sensitivity of 89.0% and a specificity of 90.0% in the diagnosis of PDAC (137). Zou et al. evaluated *EPHA2* mRNA expression levels in pancreatic adenocarcinoma (PAAD) using data from publicly available databases. The results showed that PAAD tumor tissues have aberrantly high EphA2 expression levels compared to normal tissues. Furthermore, a significant correlation was observed between EphA2 expression and the pathological stage of this tumor, with high EphA2 expression being correlated with a poor prognosis. These data also suggested that EphA2 has an essential function and clinical prognostic value in PDAC (138).

Liu et al. transfected Panc-1 and BxPC-3 cells with a small interfering RNA (siRNA) to knock down the expression of *EPHA4.* The results showed that knockdown of *EPHA4* by siRNA inhibits motility and invasion of PC cells. In addition, the gelatin zymography assay showed that *EPHA4* can regulate matrix metalloproteinase (MMP)-2 activity. Also, knockdown of *EPHA4* increased the expression of E-cadherin as well as decreased the expression of Snail. The results of the study suggest that *EPHA4* may promote the motility and invasion of PC cells by up-regulating MMP-2 and Snail, as well as down-regulating E-cadherin, and may become a useful target for the treatment of this tumor (139).

Another study explored the association between EphA4 expression and the prognosis of patients with PDAC. It also evaluated the cytostatic effect of 2,5-dimethylpyrrolyl benzoic acid (compound 1), a small molecule EphA4 blocker, on PDAC cells. As a result, patients with EphA4-positive PDAC were found to have significantly lower overall survival compared to EphA4-negative patients. The results demonstrated cytostatic efficacy in PDAC cells expressing EphA4, both *in vitro* and in orthotopic pancreatic cancer models, by suppressing the phosphorylation of EphA4 and Akt, and inducing apoptosis. Thus, 2,5-dimethylpyrrolyl benzoic acid could be a promising therapeutic agent for patients with this cancer (140).

The IHC expression of EphA1, A2, A4, A5, and A7 and the staining intensity were evaluated in tumor samples from 67 patients with PDAC. These receptors were abundantly expressed in this tumor. EphA1 staining intensity was significantly associated with tumor size and histopathological stage. EphA2 expression was significantly associated with patient age, while EphA4 and EphA5 with tumor proliferative capacity. In addition, patients with PDAC with moderate/intense EphA5 or EphA7 staining had significantly shorter survival times compared to those with weak/negative staining (17).

*EPHA10* silencing reduced the proliferation, migration, and adhesion of MIA PaCa‐2 and AsPC‐1 pancreatic cancer cells. Importantly, overexpression and silencing of this ligand respectively increased and decreased the weight, volume, and number of Ki‐67‐positive proliferating cells in MIA PaCa‐2 xenograft tumors. Further, EphA10 expression was positively correlated with invasion and gelatin degradation in these cells. In addition, the expression of *EPHA10* increased the phosphorylation of ERK, JNK, AKT, FAK, and NF‐κB, which are important for cell proliferation, survival, adhesion, migration, and invasion. Thus, EphA10 plays a pivotal role in the tumorigenesis of pancreatic epithelial cells and would be a novel therapeutic target for pancreatic cancer (141).

In summary, EphA2, EphA4, EphA5, and EphA10 play key roles in PDAC progression. EphA2 is overexpressed and linked to poor prognosis, making it a potential diagnostic and therapeutic target. EphA4 promotes cell motility and invasion, and its inhibition could reduce tumor progression. EphA5 and EphA7 are associated with tumor growth, with higher expression correlating with shorter survival. EphA10 promotes cell proliferation, survival, and invasion, making it a promising target for therapy. Overall, these Eph receptors act as tumor promoters and represent potential targets for PDAC treatment.

##### 3.4.2.2 Ephrin-B and EphB

The EphB4/ephrinB2 signaling pathway has been extensively investigated within the class B EPH/ephrin system (142). The gene and protein expression, *in vitro* and *in vivo*, was found to be significantly increased in cancer tissues and was associated with the clinical stage of PDAC and Ki67 expression. In addition, the downregulation of *EFNB2* decreased PDAC cell migration and invasion by blocking EMT. These findings suggest that *EFNB2* may participate in PDAC development by promoting cell proliferation, migration, and invasion, and may become a potential target for diagnosis and treatment of this tumor (143).

Overexpression of the *EPHB2* and a modest overexpression of *EFNB2* was also observed in 44% of 46 PDAC primary tumors and corresponded to abdominal and/or back pain. The results of the present study suggest that an increased level of ephrin-B2, in the presence of a high expression of *EFNB2*, leads to a more aggressive tumor phenotype. In summary, *EPHB2* can be used as a prognostic factor for this cancer (144).

Inhibition of EphB4/ephrinB2, *in vitro* and in a mouse xenograft model, was associated with an improvement of antitumor responses, particularly when combined with radiotherapy (RT), which resulted in notable reductions in tumor growth in cases of PDAC (145). Other *in vivo* findings from orthotopic xenografts demonstrated improved inhibition of tumor growth when *EPHB4* inhibition was combined with Gemcitabine chemotherapy (142).

The existing literature provides limited insights into the Eph/Ephrin system related to PanNETs. The MAPK pathway initiates ERK activation, a process implicated in cancer initiation via the overexpression of RTKs, such as Eph receptors (**Figure 1**) (146). Additionally, EphA RTK activation has been linked to the suppression of insulin secretion in pancreatic islets (147). An *in vitro* and *in vivo* study explored the potential of direct EphA signaling to increase glucose-stimulated insulin secretion in pancreatic islets, with results indicating that inhibition of Eph, in particular EphA5, improves glycemic control (148). These findings are promising for the treatment of metabolic disorders, particularly in insulin-producing NETs.

## 4 Conclusion and future directions

The Eph/Ephrin system continues to be a significant subject of ongoing research due to its varying roles in different cancers. Higher levels of EphA2 and ephrinA1 are associated with poorer survival rates in esophageal and gastric cancers. Among hepatocellular carcinoma, ephrinA1 also stands out as a significant biomarker, correlating with increased levels of alpha-fetoprotein and being involved in tumor growth and metastasis. Similarly, in pancreatic ductal adenocarcinoma, EphA2 is associated with a poor prognosis and is considered a sensitive diagnostic biomarker. Additionally, the inhibition of Eph receptors in pancreatic neuroendocrine tumors has been shown to reduce insulin secretion, making them a potential therapeutic target. Although Eph receptors are promising targets for cancer therapy and biomarker development, their effects can vary depending on the type of tumor. The development of anti-cancer therapies targeting the inhibition of small molecules holds great potential for the future. In this context, the advancement of clinical trials is crucial to translate these findings into effective treatments.
